# An ankylosing spondylitis-associated genetic variant in the *IL23R-IL12RB2* intergenic region modulates enhancer activity and is associated with increased Th1-cell differentiation

**DOI:** 10.1136/annrheumdis-2015-208640

**Published:** 2016-02-25

**Authors:** Amity R Roberts, Matteo Vecellio, Liye Chen, Anna Ridley, Adrian Cortes, Julian C Knight, Paul Bowness, Carla J Cohen, B Paul Wordsworth

**Affiliations:** 1 Nuffield Department of Orthopaedics, Rheumatology and Musculoskeletal Sciences, Botnar Research Centre, University of Oxford, Oxford, UK; 2 Division of Clinical Neurology, Nuffield Department of Clinical Neurosciences, John Radcliffe Hospital, University of Oxford, Oxford, UK; 3 Wellcome Trust Centre for Human Genetics, Roosevelt Drive, University of Oxford, Oxford, UK

**Keywords:** Ankylosing Spondylitis, Gene Polymorphism, T Cells

## Abstract

**Objectives:**

To explore the functional basis for the association between ankylosing spondylitis (AS) and single-nucleotide polymorphisms (SNPs) in the *IL23R*-*IL12RB2* intergenic region.

**Methods:**

We performed conditional analysis on genetic association data and used epigenetic data on chromatin remodelling and transcription factor (TF) binding to identify the primary AS-associated *IL23R-IL12RB2* intergenic SNP. Functional effects were tested in luciferase reporter assays in HEK293T cells and allele-specific TF binding was investigated by electrophoretic mobility gel shift assays. *IL23R* and *IL12RB2* mRNA levels in CD4+ T cells were compared between cases homozygous for the AS-risk ‘A’ allele and the protective ‘G’ allele. The proportions of interleukin (IL)-17A+ and interferon (IFN)-γ+ CD4+ T-cells were measured by fluorescence-activated cell sorting and compared between these AS-risk and protective genotypes.

**Results:**

Conditional analysis identified *rs11209032* as the probable causal SNP within a 1.14 kb putative enhancer between *IL23R* and *IL12RB2.* Reduced luciferase activity was seen for the risk allele (p<0.001) and reduced H3K4me1 methylation observed in CD4+ T-cells from ‘A/A’ homozygotes (p=0.02). The binding of nuclear extract to the risk allele was decreased ∼3.5-fold compared with the protective allele (p<0.001). The proportion of IFN-γ+ CD4+ T-cells was increased in ‘A/A’ homozygotes (p=0.004), but neither *IL23R* nor *IL12RB2* mRNA was affected.

**Conclusions:**

The *rs11209032* SNP downstream of *IL23R* forms part of an enhancer, allelic variation of which may influence Th1-cell numbers. Homozygosity for the risk ‘A’ allele is associated with more IFN-γ-secreting (Th1) cells. Further work is necessary to explain the mechanisms for these important observations.

## Introduction

Ankylosing spondylitis (AS) is the prototypic spondyloarthropathy (SpA), characterised by prominent axial skeletal involvement and enthesitis.^1^ Genome-wide association studies (GWAS) have clearly demonstrated the polygenic nature of AS.^2–4^ Further, many of the genes that have been implicated are also associated with conditions like inflammatory bowel disease (IBD) and psoriasis that occur much more commonly in individuals with AS than the general population.^5^ ^6^ Of these, *IL23R* (encoding the specific portion of the heterodimeric interleukin (IL)-23 receptor) was the first to be associated with AS.^7^ More than 40 loci have now been implicated in AS, several of which (eg, *IL23R, IL12B, IL6R, TYK2, IL27R, IL1R2, IL1R1* and *STAT3*) potentially affect IL-23-driven pro-inflammatory pathways.^2–4^ ^8^ ^9^ The importance of these pathways is further highlighted by the potential role of IL23R-expressing cells at the entheses in murine models of SpA.^10^ The primary *IL23R* association with AS (also psoriasis and IBD) is with *rs11209026*, a missense variant (Arg381Gln) in the cytoplasmic tail, which alters IL-23R signalling.^7^ ^11^ ^12^ In addition, a second independent association signal has been identified in the intergenic region downstream of *IL23R* and upstream of *IL12RB2* (encoding the 130kD β2 chain specific to the IL-12 receptor).^3^ This second signal is also associated with IBD.^5^ Currently, the mechanism underlying the latter association is unknown. In this study, we have identified a putative regulatory element (PRE) between *IL23R* and *IL12RB2*. We have then investigated the impact of AS-associated single-nucleotide polymorphisms (SNPs) on the function of this PRE.

## Methods

### Identification of a PRE

We used a combination of published AS GWAS data^3^ and epigenetic data from the ENCODE^13^ and Roadmap Epigenomics Projects,^14^ to identify a 1.14 kb PRE between *IL23R* and *IL12RB2,* including the AS-associated SNPs *rs11209032* and *rs6677188.* The epigenetic data included DNase I hypersensitivity sites, transcription factor (TF) binding sites and histone modifications.

### Patients with AS

All patients in these studies fulfilled the modified New York AS criteria^15^ or ASAS axial SpA imaging criteria.^16^ Following informed consent, blood samples for the functional studies (below) were obtained from patients.

#### IFN-γ+ and IL-17A+ T-cell FACS analysis

Blood samples were obtained from 52 biologic-naive AS cases (mean age 42 years±SD 12.3). The mean Bath AS disease activity index (BASDAI) was 4.6±SD 2.2.

#### Gene expression

Blood samples were obtained from 12 AS cases (mean age 61.5 years±SD 12.6). The mean BASDAI was 3.4 (±SD 1.7) and mean C reactive protein 7.1 mg/L (±SD 6.4). Only nine were currently taking non-steroidal anti-inflammatory analgesics, and none were taking corticosteroids or other immunomodulatory drugs.

### Genotyping

Historical typing data from previously published AS Immunochip study^3^ were used if available or were obtained using TaqMan Genotyping Assay (Life Technologies, Paisley, UK) to assign SNP genotypes. Where required, DNA was extracted from peripheral blood mononuclear cells (PBMCs) using the QIAGEN AllPrep DNA/RNA Mini Kit (QIAGEN).

### CD4+ T-cell isolation

CD4+ T-cells were isolated from PBMCs using the negative selection CD4+ T-cell Isolation kit (Miltenyi, Bisley, Surrey, UK). CD4+ T-cells were plated for 4 h/overnight in Roswell Park Memorial Institute supplemented with 10% fetal bovine serum before harvesting for experiments. Cell viability was checked with trypan blue or fluorescence-activated cell sorting (FACS) analysis.

### FACS analysis

PBMCs were isolated by density gradient centrifugation using Histopaque (Sigma, Dorset, UK), frozen and stored in liquid nitrogen before staining. Intracellular cytokine staining of Th17 and Th1-cells was carried out using BD Cytofix/Cytoperm kit (BD Bioscience, Oxford, UK). Cells were stimulated with 100 ng/mL phorbol 12-myristate 13-acetate (PMA) (Sigma, Dorset, UK) and 1 μg/mL ionomycin (Sigma, Dorset, UK) for 4 h in the presence of Golgi STOP and Golgi plug. After surface staining using CD3-BV605, CD4-APC and CD8-BV510 antibodies (Biolegend, London, UK), cells were fixed and permeabilised, then stained with IL-17A-FITC (eBiosciences, Ireland, UK) and interferon (IFN)-γ-AF700 (Biolegend). Dead cells were excluded using Fixable Viability Dye eFluor 780 (eBiosciences). Representative FACS plots of the gating strategy and intracellular staining are shown in online supplementary figure S1.

10.1136/annrheumdis-2015-208640.supp3 Supplementary figures

### Electrophoretic mobility shift assay

Nuclear extract from HEK293 cells (human embryonic kidney cell line) was purchased from Active Motif (Belgium, Germany). Electrophoretic mobility shift assays (EMSAs) were performed with LightShift Chemiluminescent EMSA Kit (Thermo Scientific, Waltham, USA) using 5 μg of nuclear extract, and 10 fmol biotin labelled double-stranded oligonucleotides (50 bp fragment—Eurofins, Wolverhampton UK). The sequences of the synthetic single-stranded oligonucleotides used in the construction of these double-stranded oligonucleotides are listed in the online supplementary methods. Single-stranded biotinylated oligonucleotides were mixed and annealed at room temperature for 1 h. Unlabelled competitor probes were in 100-fold excess. EMSAs were performed according to standard protocol (Thermo Scientific). The involvement of TWIST1 in these DNA-protein complexes was investigated by including TWIST1 antibody (ab50877—Abcam, Cambridge, UK). The results were confirmed in five independent experiments. Band intensity from these five experiments was measured by ImageJ Launcher (V.1.4.3.67). A detailed protocol is available in online supplementary methods.

10.1136/annrheumdis-2015-208640.supp1 Supplementary data

### Luciferase reporter assay

The 1.14 kb PRE sequence was amplified from genomic DNA and cloned into TA cloning kit pCR2.1 vector (Invitrogen, Paisley, UK). It was subcloned into pGL4.23 [luc2/minP] reporter vector (Promega, Madison, USA) at the *Sac*I/*Xho*I restriction sites upstream of the minimal promoter necessary to drive the luciferase reporter gene (primer sequences available on request). Point mutations corresponding to genetic variants (G/A) of *rs11209032* were introduced using the QuikChange II XL Site-Directed Mutagenesis Kit (Agilent, Santa Clara, USA). Luciferase reporter assay details are available in online supplementary methods.

### Quantitative real-time RT-PCR

RNA was isolated from CD4+ T-cells from patients of different genotypes (PMA and ionomycin stimulated; overnight). RNA isolation was performed using the Allprep DNA/RNA Mini kit (QIAGEN) and cDNA synthesis (for 500 ng RNA) was prepared with Superscript III from Invitrogen. A final concentration of 5 ng was used in quantitative PCR (qPCR), which was performed with the ABI ViiA7 PCR instrument (Applied Biosystems, Paisley, UK) using SYBR Master mix (Applied Biosystems) with evaluation of dissociation curves. mRNA levels of each gene were quantified using the DDCt method and expressed relative to *β-actin*. For each gene, a TaqMan Gene Expression Assay was used (Life Technologies—according to the manufacturer's instructions); *IL23R* (Hs00332759_m1), *IL12RB2* (Hs00155486_m1), *IFNG* (Hs00989291_m1), *IL10* (Hs00961622_m1) and *β-actin* (Hs01060665_g1). *H19* primers were purchased from Diagenode (Liege, Belgium).

### Statistical analysis

Association data for genotyped SNPs were obtained on the subset of AS cases of white British ancestry and white British controls from Immunochip GWAS.^3^ To evaluate the presence of independent effects on genetic susceptibility at the *IL23R-IL12RB2* intergenic region, we performed conditional analysis on 4230 AS cases and 9700 matched controls, as previously described.^3^ Association analysis was performed using the logistic regression function in PLINK, V.1.90, accounting for population structure with 10 principal components.^17^ One-way analysis of variance and two-tailed Student's t test were used to determine statistical significance using GraphPad Prism (V.5.03).

## Results

### Identification of a PRE within *IL23R-IL12RB2* intergenic region associated with AS

**Figure 1:**
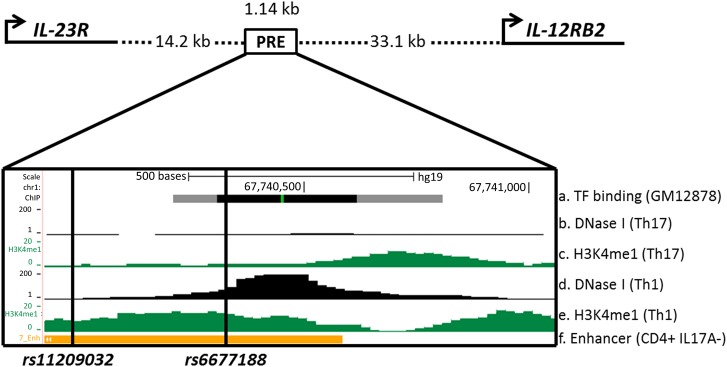
Epigenetic and transcriptional landscape of the 1.14 kb putative regulatory element (PRE) containing *rs11209032* and *rs6677188* downstream of *IL23R*. Cartoon representation of *IL23R* and *IL12RB2* promoter and PRE location (Chr1:67739940–67741075). ENCODE and Roadmap data: (A) Transcription factor (TF) binding sites (grey/black box) in lymphoblastoid cell line GM12878. (B) DNase I hypersensitivity in Th17-cells. (C) H3K4me1 methylation in Th17-cells. (D) DNase I hypersensitivity in Th1-cells. (E) H3K4me1 methylation in Th1-cells. (F) Enhancer chromatin state in CD4+ CD25− interleukin (IL)-17A− T-cells (PMA and ionomycin-stimulated).

We identified a PRE (Chr1:67739940–67741075) located 14.2 kb downstream of *IL23R* and 33.1 kb upstream of *IL12RB2* (figure 1). This PRE overlaps a region reported in the Immunochip study to be independently associated with AS after conditioning on association at the primary *rs11209026* coding SNP.^3^ This region is DNase I hypersensitive (in Th1-cells, but not Th17-cells) and exhibits TF binding (lymphoblastoid cell line; GM12878) and enhancer-associated H3K4me1 methylation (CD4+ CD25− IL-17A− T-cells; PMA and ionomycin-stimulated).^1^ ^3^ ^14^

### Conditional analysis identifies *rs11209032* as a candidate casual variant

The PRE contains 10 SNPs, 3 of which show strong or weak association with AS; *rs11209032* (p=5.8×10^−16^), *rs80216366* (p=4.9×10^−21^) and *rs6677188* (p=2×10^−4^). Conditional analysis previously performed on the primary *IL23R* coding SNP *rs11209026* revealed no residual association at *rs80216366* (p=0.4), indicating that this association is due to linkage disequilibrium (LD) with *rs11209026.*^3^ The associations at *rs11209032* (p=1.5×10^−9^) and *rs6677188* (p=9.3×10^−7^) were robust to conditioning on *rs11209026*, revealing a second region of independent association.^3^ We then performed further conditional analysis on *rs11209032* or *rs6677188*, respectively, and found that the association at *rs6677188* disappeared after conditioning on *rs11209032*, indicating that this association was due to LD with *rs11209032* (table 1). In contrast, the strong association with *rs11209032* was retained (p=8.1×10^−12^) after conditioning on *rs6677188*, thereby establishing the primacy of the *rs11209032* association with AS in this region. However, a statistical association does not necessarily guarantee functionality. Initial interrogation of publicly available datasets suggested that *rs6677188* might actually be more functionally relevant than *rs11209032* (despite the genetic association data above). *rs6677188* overlaps DNase I hypersensitivity (in Th1-cells) and TF binding sites (in GM12878 cells), whereas *rs11209032* lies on the border of these sites (see figure 1). In the following functional study, we therefore analysed both SNPs.

**Table 1:** Conditional analysis of single-nucleotide polymorphism (SNP) associations at IL23R-IL12RB2 intergenic region

Position*	Conditional SNP	Risk/protective	SNP	p Value	OR	RAF (case/control)	LD (r^2^/D′) with conditional SNP
Chr1:67706208	*rs11209026*†	G/A	*rs11209032*	1.5×10^−9^	1.17	0.96/0.93	0.03/0.97
			*rs80216366*	0.4	1.11		0.83/0.94
			*rs6677188*	9.3×10^−7^	0.91		0.01/0.87
Chr1:67740342	*rs11209032*	A/G	*rs11209026*	9.5×10^−14^	0.62	0.37/0.33	0.03/0.97
			*rs80216366*	3.6×10^−10^	0.69		0.03/0.98
			*rs6677188*	0.2	1.04		0.17/1
Chr1:67740653	*rs6677188*	A/T	*rs11209026*	3×10^−18^	0.58	0.24/0.25	0.01/0.87
			*rs11209032*	8.1×10^−12^	1.23		0.17/1
			*rs80216366*	1.3×10^−14^	0.63		0.02/1

*NCBI Build 37 human genome coordinates. †Data from ref. 3

Chr., chromosome; LD, linkage disequilibrium; RAF, risk allele frequency.

### Homozygosity for the AS-risk allele is associated with increased Th1-cell frequencies

**Figure 2:**
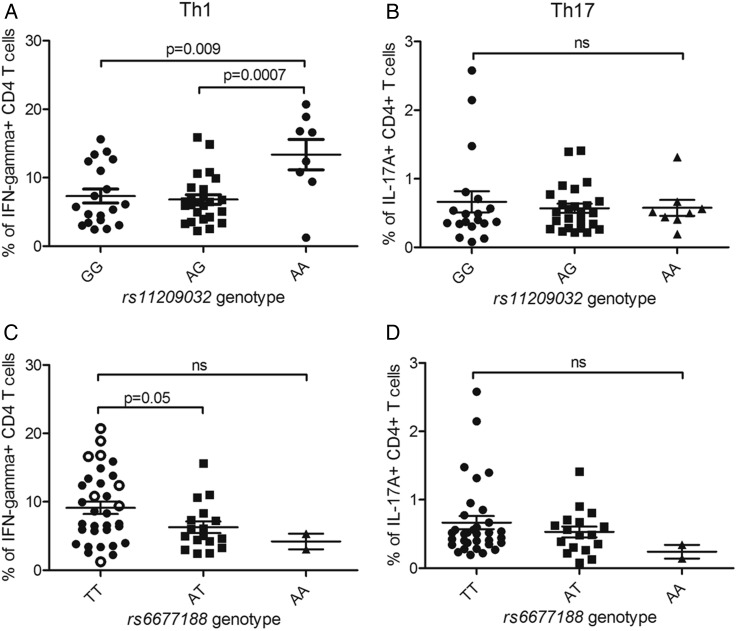
Homozygosity for the ankylosing spondylitis (AS)-risk allele at *rs11209032* is associated with increased Th1-cell frequencies. (A) The % of interferon (IFN)-γ+ CD4+ T-cells in patients with AS of each genotype at *rs11209032* (19 GG, 25 GA, 8 AA). (B) The % of interleukin (IL)-17A+ CD4+ T-cells in patients with AS of each genotype at *rs11209032* (19 GG, 25 GA, 8 AA). (C) The % of IFN-γ+ CD4+ T-cells in patients with AS of each genotype at *rs6677188* (33 TT, 17 TA, 2 AA). Patients ‘A/A’ at *rs11209032* are highlighted by open circles. (D) The % of IL-17A+ CD4+ T-cells in patients with AS of each genotype at *rs6677188* (33 TT, 17 TA, 2 AA). The percentage of cells is expressed as mean±SEM. Student's t test was used.

We sought to determine the mechanisms by which *rs11209032* or *rs6677188* affect AS susceptibility. The PRE containing *rs11209032* and *rs6677188* lies between *IL23R* (expressed on Th17-cells) and *IL12RB2* (expressed on Th1-cells). Therefore, we measured the frequencies of Th17- and Th1-cells from patients with known SNP genotypes. Th17-cell and Th1-cell frequencies were measured by FACS for IL-17A+ and IFN-γ+ CD4+ T-cells, respectively (figure 2). A significant increase in the percentage of IFN-γ+ CD4+ T-cells was observed in patients homozygous for the ‘A’ (risk) allele at *rs11209032* (figure 2A, p<0.01), but not for IL-17A+ CD4+ T-cells (figure 2B). The ‘T/T’ genotype at *rs6677188* is weakly associated with an increased percentage of IFN-γ+ CD4+ T-cells (p=0.05, figure 2C), but not IL-17A+ CD4+ T-cells (figure 2D). However, this observed increase in the percentage of IFN-γ+ CD4+ T-cells apparently associated with *rs6677188* is actually secondary to the association with the *rs11209032* ‘A’ (risk) allele, which is in complete LD with the *rs6677188* ‘T’ allele. If the *rs11209032* ‘A/A’ homozygotes are excluded from the *rs6677188* analysis (open circles in figure 2C), the apparent difference disappears. There was no correlation between the frequency of double-positive IL-17A+/IFN-γ+ CD4+ T-cells and the SNP genotype (see online supplementary figure S2). Overall, these results are consistent with the conditional analysis in suggesting that *rs11209032* is the most probable causal variant (through effects on Th1-cell differentiation). Support for an effect in Th1-cells comes from publicly available data sets that show the presence of DNase I hypersensitivity in Th1-cells within the PRE, but not in Th17-cells (see figure 1). Consequently, all the subsequent experiments focused on *rs11209032* alone.

### Differential binding of nuclear extract at *rs11209032*

**Figure 3:**
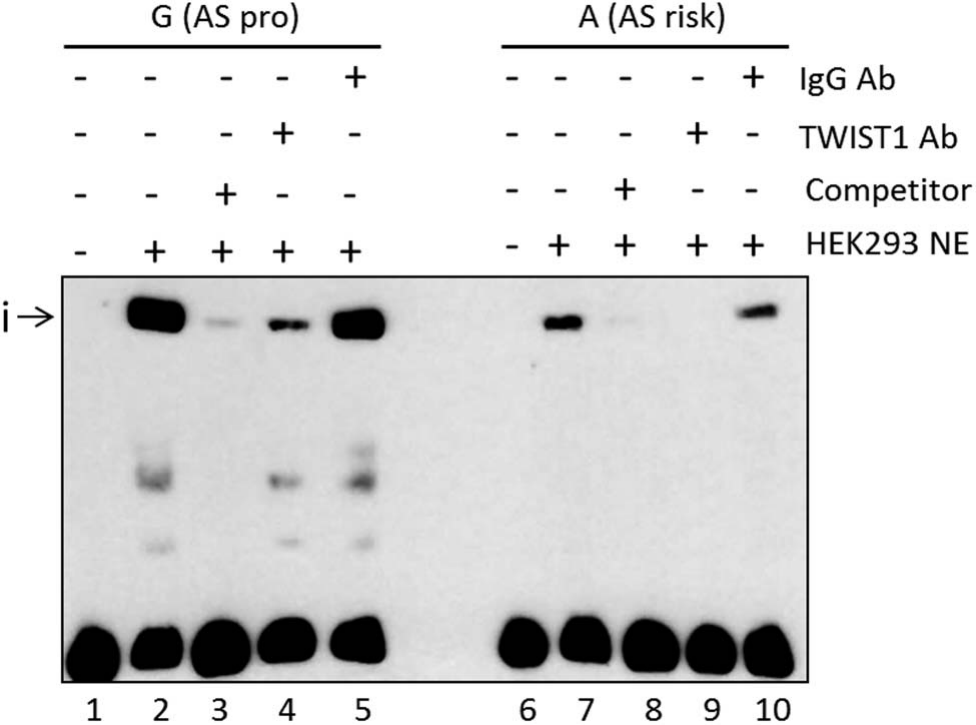
The ankylosing spondylitis (AS)-risk allele at *rs11209032* alters DNA-protein complex formation. Chemiluminescent electrophoretic mobility shift assay (EMSA) showing complex formation (i) after addition of HEK293 nuclear extract (lanes 2 and 7), and competition with 100-fold excess of unlabelled probes (lanes 3 and 8). TWIST1 antibody addition leads to a reduction or inhibition of the complex (i) (lanes 4 and 9, respectively). Addition of nonspecific IgG antibody (lanes 5 and 10). NE, nuclear extract; pro, protective. This result was confirmed in five independent experiments.

We investigated the mechanism for the increase in IFN-γ+ CD4+ T-cells (above) by looking for effects of *rs11209032* on TF binding. EMSAs were performed using nuclear extract from HEK293 cells (human embryonic kidney cell line). The addition of nuclear extract to a 50mer DNA probe containing *rs11209032* revealed a DNA-protein complex (i), with ∼3.5-fold lower binding to the ‘A’ (risk) allele (figure 3, p<0.001). Binding to the ‘G’ (protective) allele was outcompeted by 100-fold excess of unlabelled ‘G’ (figure 3 and online supplementary figure S3A).

A search for TF binding consensus sequences that directly overlap *rs11209032* revealed a CTCF binding motif (in lymphoblastoid cell line GM12878).^18^ However, *rs11209032* genotype had no influence on binding (data not shown). The consensus sequence of the Th1 transcriptional repressor TWIST1 (5′-NCANNTGN-3′) is located 1 bp 3′ of the *rs11209032* SNP. HEK293 cells are a good source of TWIST1 protein (see online supplementary figure S3B). Addition of TWIST1 antibody to the EMSA reduced/inhibited the formation of the DNA-protein complex for both alleles (figure 3).

### *rs11209032* alters levels of H3K4me1 methylation

**Figure 4:**
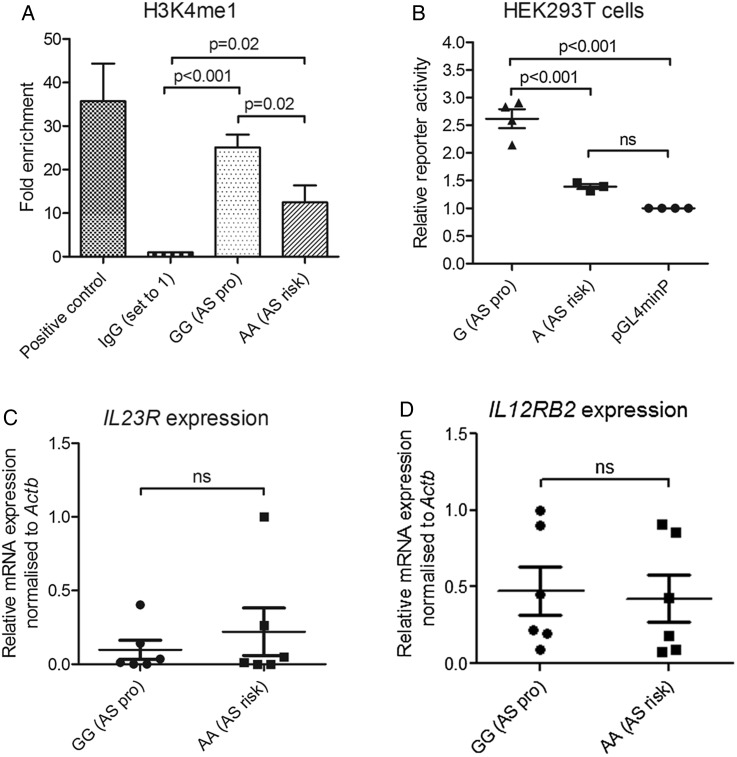
Homozygosity for the ankylosing spondylitis (AS)-risk allele at *rs11209032* is associated with reduced enhancer activity without altering mRNA levels of *IL23R* or *IL12RB2.* (A) H3K4Me1 methylation ChIP-qPCR assessed at the *rs11209032* locus in CD4+ T cells (PMA and ionomycin-stimulated) from three ‘G/G’ and three ‘A/A’ patients. The fold enrichment is expressed as mean±SEM for each patient in triplicate. Positive control is *IL10* enhancer. Student's t test was used. (B) The transcriptional activity of *rs11209032* compared with minP (set to 1) was measured by luciferase reporter assays in HEK293T cells. The values of relative luciferase activity are expressed as mean±SEM of three or four repeat experiments each done in triplicate. One-way analysis of variance was used. (C) Relative amount of *IL23R* mRNA in primary CD4+ T-cells (PMA and ionomycin-stimulated) from 6 GG and 6 AA patients (normalised against *β-actin*). mRNA levels are expressed as mean±SEM. Student's t test was used. (D) Relative amount of *IL12RB2* mRNA in primary CD4+ T-cells (PMA and ionomycin-stimulated) from 6 GG and 6 AA patients (normalised against *β-actin*). mRNA levels are expressed as mean±SEM. Student's t test was used. minP, minimal promoter.

Publicly available data show that the region overlapping *rs11209032* is enriched for enhancer-associated H3K4me1 methylation in Th1-cells.^14^ To investigate enhancer activity ex vivo, we assessed the levels of H3K4me1 methylation at *rs11209032* by ChIP-qPCR. Homozygosity for the ‘A’ (risk) allele correlated with reduced H3K4me1 abundance in CD4+ T cells from patients with AS, whereas the level in ‘G/G’ homozygotes was similar to the positive control (*IL10* enhancer) (figure 4A, p=0.02).

### The AS-risk allele at *rs11209032* shows reduced reporter activity

We used luciferase reporter assays to confirm the effect of *rs11209032* genotype on enhancer activity. Luciferase reporter assays were performed in HEK293T cells transfected with pGL4.23 plasmids containing the minimal promoter (minP) and the 1.14 kb PRE sequence (see figure 1) with either the ‘A’ (risk) allele or the ‘G’ (protective) allele. The ‘G’ (protective) allele showed significantly increased reporter activity above the minP level of 1 (figure 4B, p<0.001), which provides further support to PRE having enhancer activity. Interestingly, the ‘A’ (risk) allele significantly reduced this activity compared with the protective allele (p<0.001).

### No correlation between *rs11209032* genotype and *IL23R* or *IL12RB2* mRNA levels

We next assessed the effect of the *rs11209032* genotype and reduced enhancer activity on expression levels of the nearby genes. Analysis of *IL23R* and *IL12RB2* expression in CD4+ T-cells isolated from patients with AS (PMA and ionomycin-stimulated overnight, cell viability≥71%) with different *rs11209032* genotypes (6 GG and 6 AA) showed no significant effect on mRNA levels (figure 4C,D). There was also no correlation between disease activity and mRNA levels for either gene (data not shown).

## Discussion

We have clearly shown the primacy of the *rs11209032* SNP in a potentially important regulatory region between *IL23R* and *IL12RB2* associated with AS. The *rs11209032* AS-risk ‘A’ allele influences the formation of a TF complex, which includes TWIST1. Luciferase reporter assays showed an effect of the AS-risk allele on transcription but, somewhat surprisingly, we could not detect an effect on expression of the two nearby genes *IL23R* or *IL12RB2*. Our experiments on gene expression were not highly powered and the absence of definitive high and/or low expresser cell type controls somewhat hindered their interpretation. Overall, these data suggest that this regulatory element does not influence the expression of the neighbouring genes. However, we have not formally excluded the possibility it could modulate the function of these genes under different stimulation conditions from those that we have used or according to the differentiation status of the cells. It is also possible that there may be other regulatory elements in this region, which we have not yet identified that could influence these genes. These possibilities will be investigated systematically in future studies.

AS is a complex polygenic disease showing multiple genetic associations with the IL-23 pathway. Here, we show that homozygosity for the AS-risk ‘A’ allele of an SNP in the *IL-23R-IL12RB2* intergenic region is associated with increased Th1-cell numbers. We have not been able to define the regulatory mechanism(s) involved precisely. Our EMSA data show that TWIST1 binds to the region containing *rs11209032*. TWIST1 is reported to be a transcriptional repressor of Th1 gene expression and cytokine production, specifically reducing *Ifn-γ* expression in mouse cells.^19^ It is, therefore, possible that altered binding of TWIST1 to the putative enhancer may contribute to the findings reported here. We now intend to investigate more deeply the involvement of TWIST1 and the full nature of the DNA-protein complex with *rs11209032*, which is reduced in the presence of the risk ‘A’ allele, to look for a potential network of genes that might be regulated at least in part by this SNP.

Inspection of 2 Mb of flanking sequence around *rs11209032* reveals no obvious Th1-related genes. However, since only 27% of distal regulatory elements are reported to interact with the nearest promoter, the target of an SNP disease association is often not with the nearest gene and may extend over several megabases.^20^ ^21^ Substantial work may be required to identify the target gene(s).

Double-positive IL-17A+/IFN-γ+ CD4+ T-cells have been reported in synovial fluid and synovial tissue of some patients with rheumatoid arthritis (RA)^22–24^ and are increased in peripheral blood from patients with AS and RA.^25^ However, we found no evidence of an influence of SNP genotype on the frequency of these cells. Previously, increased IFN-γ production has been reported in T-cells from patients with AS,^26–28^ although this is controversial because others report reduced IFN-γ levels.^29^ Here, we report a correlation of *rs11209032* genotype with the percentage of IFN-γ+ CD4+ T-cells in patients with AS. Our results highlight the potential importance of genetic/epigenetic regulation of the Th1 pathway in the pathogenesis of AS.

There are at least two distinct genetic effects arising from the vicinity of *IL23R* in AS. The first (*rs11209026*) has been previously characterised and encodes an Arg381Gln change in the cytoplasmic tail of IL-23R that reduces signalling both in healthy donors and patients with inflammatory arthritis.^11^ ^12^ ^30^ ^31^ Such protein-coding changes constitute only a minority of genetic associations with common diseases, perhaps indicative of their relatively pronounced functional effects. In contrast, more subtle effects on gene expression or protein binding, as represented by the *IL23R-IL12RB2* association we describe here, are probably more common and may act synergistically.^32^ Our study highlights the power of conditional analysis to identify the primary genetic disease associations definitively. On first inspection, *rs6677188*, which lies in the same associated region as *rs11209032*, appears somewhat more likely to be functionally relevant because it is closer to the local peak of DNase I hypersensitivity. However, the conditional analysis unequivocally demonstrated the primacy of the *rs11209032* association since the apparent association with *rs6677188* disappeared after conditioning on *rs11209032*.

Genetic associations with disease identified by GWAS can potentially identify new drug targets even where the strength of the association is relatively weak. In general, polymorphisms influencing gene expression are more likely to be implicated in polygenic diseases like AS. Additive influences arising from numerous SNPs in the IL-23 pathway, which alter the effector functions of Th1-cells and Th17-cells in patients with SpA, have been described^32^ but the full complexity of the regulation of these cells is only just becoming apparent.^33^

The combination of an analytical approach with a computational and experimental validation can identify important transcriptional gene networks, such as those governing the specification and function of Th1-cells in health and disease (eg, *T-bet, STAT4, RUNX3* and *TWIST1*).^19^ ^34^ A similar approach has been used to identify and explain the association between a functional polymorphism in the tumour necrosis factor receptor gene (*TNFRSF1A*) and multiple sclerosis.^35^ Further studies in AS will focus on identifying the factors contributing to the reduced enhancer activity reported here and how these factors contribute to increased Th1-cell signalling.
