# Supplementary material for: An ankylosing spondylitis-associated genetic variant in the IL23R-IL12RB2 intergenic region modulates enhancer activity and is associated with increased Th1-cell differentiation
Source: Ann Rheum Dis. 2016 Feb 25;75(12):2150–6. doi: 10.1136/annrheumdis-2015-208640 (PMC5136719; doi:10.1136/annrheumdis-2015-208640)

Supplementary Figures:

Suppl. Figure 1: Representative FACS plots of the gating strategy and intracellular staining of IL-17A+ CD4+ T-cells, IFN-γ+ CD4+ T-cells, and double-positive cells in AS patients. The numbers of IL-17A+ and IFN-γ+ cells were taken from the left and middle panels, respectively. The right panel is included here to show the gating for IL-17A/IFN-γ double-positive cells. The inclusion of murine IgG1 isotype control antibodies on several occasions confirmed the specificity of the IL-17A and IFNγ antibodies.

Suppl. Figure 2: The AS-risk allele at *rs11209032* has no effect on IL-17A+/IFN-γ+ double-positive CD4+ T cells. (A) The % of IL-17A+ IFN-γ+ CD4+ T-cells in AS patients of each genotype at *rs11209032* (19 GG, 25 GA, 8 AA). (B) The % of IL-17A+ IFN-γ+ CD4+ T-cells in AS patients of each genotype at *rs6677188* (33 TT, 17 TA, 2 AA). Percentage of cells is expressed as mean ± SEM. Student *t*-test was used.

Suppl. Figure 3: The AS-risk allele at *rs11209032* alters DNA-protein complex formation, and TWIST1 protein is expressed in HEK293 cells. (A) Chemiluminescent EMSA showing complex formation (i) after addition of HEK293 nuclear extract (lanes 2 and 6), and competition with 100-fold excess of either the unlabelled “G” or unlabelled “A” probes (lanes 3, 4 and 7, 8). (B) Western blot analysis showing TWIST1 protein expression in HEK293 cells used for EMSA and reporter assay, and in a second cell line for comparison, K562. NE: nuclear extract; pro: protective.

Supplementary Figure 1

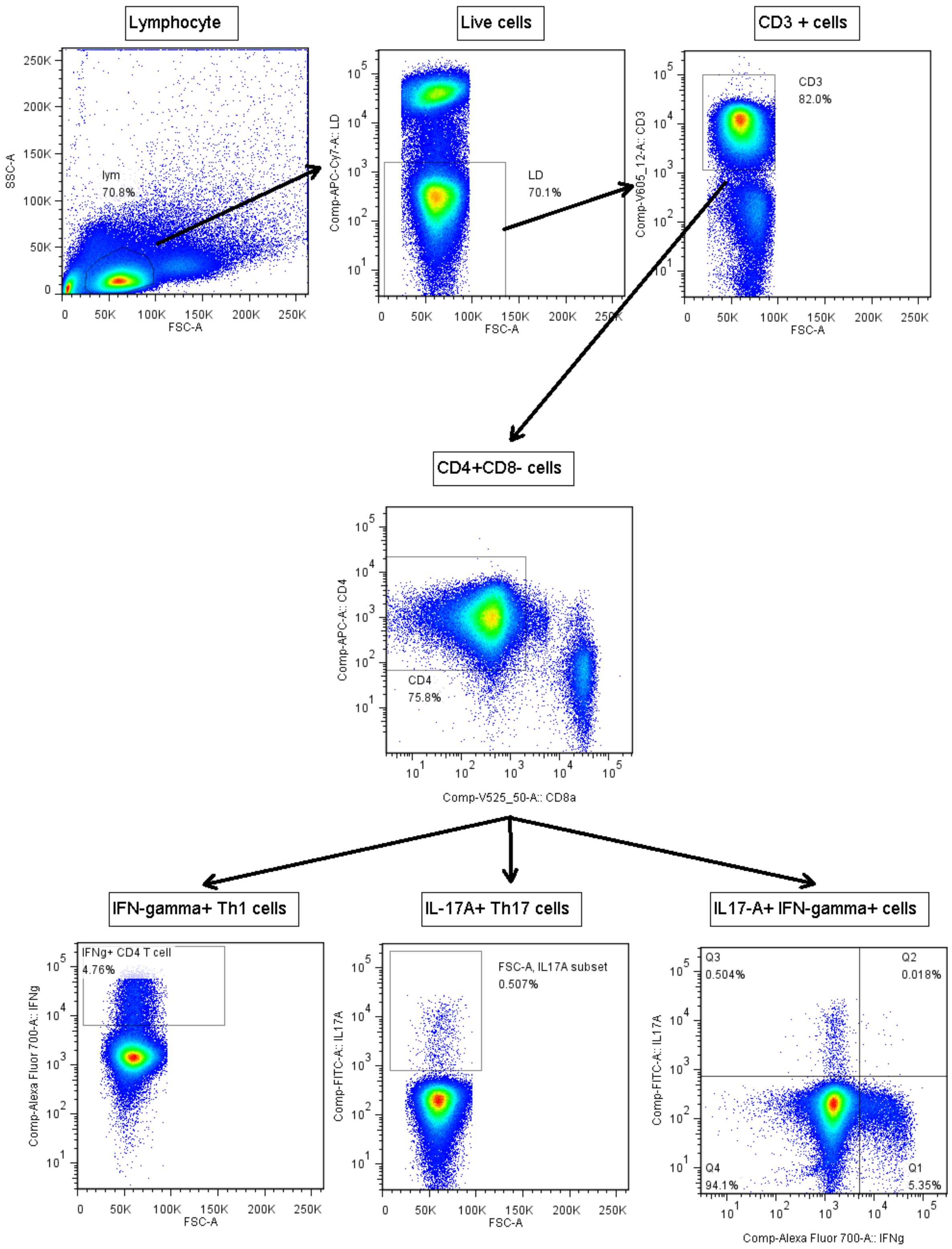

Supplementary Figure 2

A

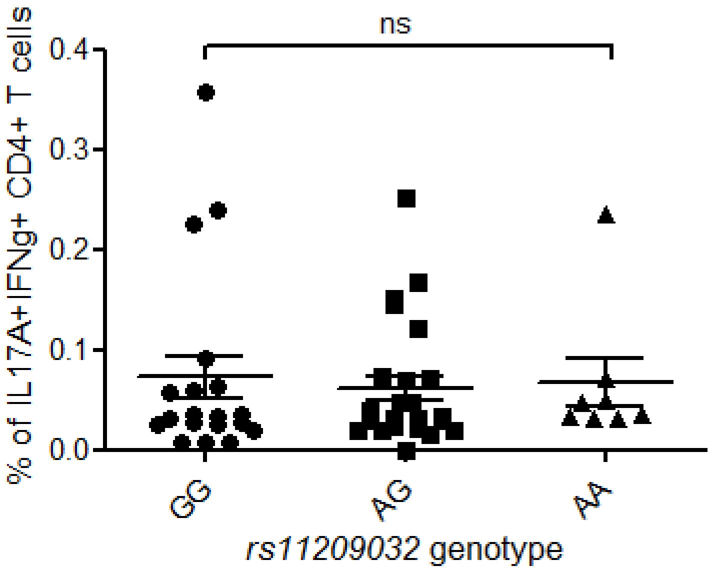

B

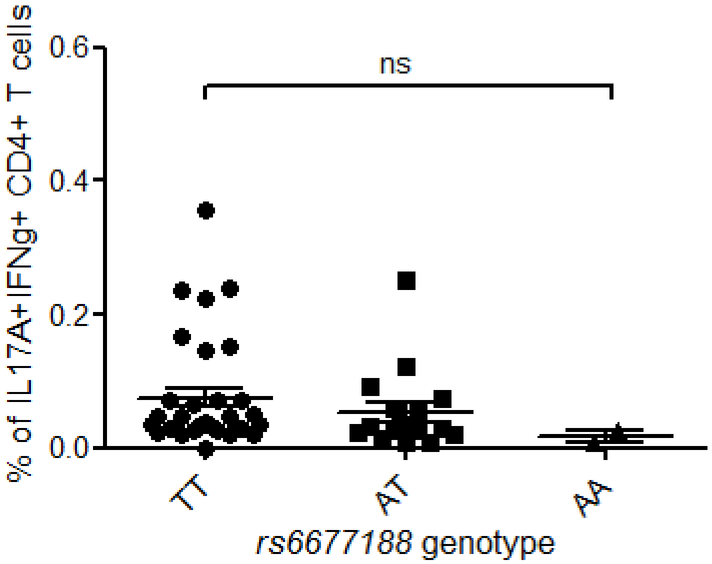

Supplementary Figure 3

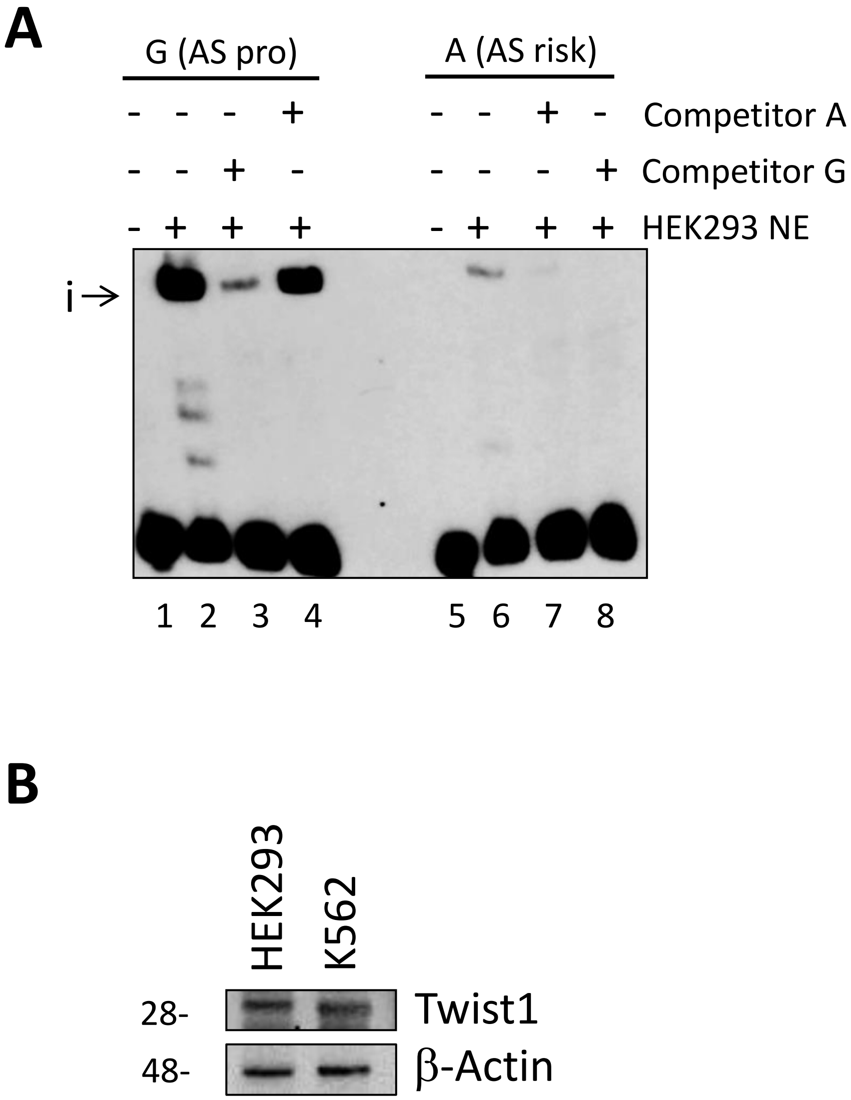

Supplement: Supplementary figures [file annrheumdis-2015-208640supp_figures.pdf]
