# Supplementary material for: An ankylosing spondylitis-associated genetic variant in the IL23R-IL12RB2 intergenic region modulates enhancer activity and is associated with increased Th1-cell differentiation
Source: Ann Rheum Dis. 2016 Feb 25;75(12):2150–6. doi: 10.1136/annrheumdis-2015-208640 (PMC5136719; doi:10.1136/annrheumdis-2015-208640)
Supplement: Supplementary data [file annrheumdis-2015-208640supp.pdf]

**SUPPLEMENTARY METHODS**

# An ankylosing spondylitis-associated genetic variant in the *IL23R-IL12RB2* intergenic region modulates enhancer activity and is associated with increased Th1-cell differentiation

Amity R Roberts[1], Matteo Vecellio[1], Liye Chen[1], Anna Ridley[1], Adrian Cortes[2,3], Julian C Knight[3], Paul Bowness[1], Carla J Cohen[1]*, B Paul Wordsworth[1]*

*BP Wordsworth and CJ Cohen are joint senior authors.

**Author affiliations**
[1]Nuffield Department of Orthopaedics, Rheumatology and Musculoskeletal Sciences, Botnar Research Centre, University of Oxford, Oxford, UK
[2]Nuffield Department of Clinical Neurosciences, Division of Clinical Neurology, John Radcliffe Hospital, University of Oxford, Oxford, UK
[3]Wellcome Trust Centre for Human Genetics, Roosevelt Drive, University of Oxford, Oxford, UK

**Address for correspondence:**
Prof BP Wordsworth
Nuffield Department of Orthopaedics, Rheumatology and Musculoskeletal Sciences,
Botnar Research Centre,
Nuffield Orthopaedic Centre
Windmill Road,
Headington,
Oxford
OX3 7LD

Email: paul.wordsworth@ndorms.ox.ac.uk
Telephone: 44 (0)1865741155

### Luciferase reporter assay

HEK293T cells were cultured in DMEM supplemented with 10% fetal bovine serum, 100 units/ml penicillin, 100 units/ml streptomycin and 2 mM L-glutamine. HEK293T cells at 15,000 cells/well in 96-well plates were co-transfected with 100 ng of pGL4 construct and 2 ng of pRL-null (Promega, Madison, USA) using GeneJuice (Merck Chemicals Ltd, Nottingham, UK). After 48 hours, luciferase activity was measured using the Dual-Luciferase assay reporter system (Promega, Madison, USA). Firefly luciferase activity was normalized relative to Renilla luciferase activity for each transfection and calculated as fold increase over pGL4.23[luc2/minP]. One-way ANOVA was used to determine significant differences between the two allelic constructs.

### Electrophoretic mobility shift assay

Single-stranded biotinilated oligonucleotides (50bp fragment - Eurofins®, Wolverhampton UK) were mixed and annealed at room temperature (RT) for 1 hour. The double-stranded oligonucleotides made of synthesised single-stranded oligonucleotides are listed below:

*rs11209032* G forward allele: 5'-TCCCTACATCACCCTCTTTGCAATGGCAGATGGAAGAA TTGGCAATAAAT-3',
*rs11209032* G reverse allele 5'-ATTTATTGCCAATTCTTCCATCTGCCATTGCAAAGAGGG TGATGTAGGGA-3',
*rs11209032* A forward allele 5'-TCCCTACATCACCCTCTTTGCAATAGCAGATGGAAGAAT TGGCAATAAAT-3'
*rs11209032* A reverse allele 5'-ATTTATTGCCAATTCTTCCATCTGCTATTGCAAAGAGGG TGATGTAGGGA-3'

Unlabelled probes were used at 100-fold excess as competitors. EMSA binding reactions contained 1x binding buffer suppled with the kit, supplemented with Poly (dI•dC) (50ng/ul). Incubations were performed at RT for 20 min. Reactions were run on pre-cast non-denaturing 6% DNA retardation gels (Invitrogen$^{TM}$, Paisley, UK) with 0.5-fold TBE running buffer (45 mM Tris, 45 mM boric acid, 1 mM EDTA, pH 8.0), chilled. The DNA was then transferred at 380 mA from the gel to nitrocellulose membrane for 60 minutes on ice and cross-linked by UV. All blocking and detection incubations were performed according to manufacturer's instructions and the membrane was exposed to X-ray film.
